# Improved Estimation of Protein-Ligand Binding Free Energy by Using the Ligand-Entropy and Mobility of Water Molecules

**DOI:** 10.3390/ph6050604

**Published:** 2013-04-26

**Authors:** Yoshifumi Fukunishi, Haruki Nakamura

**Affiliations:** 1 Molecular Profiling Research Center for Drug Discovery (molprof), National Institute of Advanced Industrial Science and Technology (AIST), 2-3-26, Aomi, Koto-ku, Tokyo 135-0064, Japan; 2 Institute for Protein Research, Osaka University, 3-2 Yamadaoka, Suita, Osaka 565-0871, Japan; E-Mail: harukin@protein.osaka-u.ac.jp

**Keywords:** protein-ligand docking, molecular dynamics simulation, protein-ligand binding free energy

## Abstract

We previously developed the direct interaction approximation (DIA) method to estimate the protein-ligand binding free energy (ΔG). The DIA method estimates the ΔG value based on the direct van der Waals and electrostatic interaction energies between the protein and the ligand. In the current study, the effect of the entropy of the ligand was introduced with protein dynamic properties by molecular dynamics simulations, and the interaction between each residue of the protein and the ligand was also weighted considering the hydration of each residue. The molecular dynamics simulation of the apo target protein gave the hydration effect of each residue, under the assumption that the residues, which strongly bind the water molecules, are important in the protein-ligand binding. These two effects improved the reliability of the DIA method. In fact, the parameters used in the DIA became independent of the target protein. The averaged error of ΔG estimation was 1.3 kcal/mol and the correlation coefficient between the experimental ΔG value and the calculated ΔG value was 0.75.

## 1. Introduction

In the pharmaceutical sciences, the protein-ligand binding free energy (ΔG) is one of the most important properties of a drug compound. Despite the development of numerous docking programs and scoring functions to estimate the ΔG [1,2,3,4,5,6,7], the typical accuracy of ΔG estimation remains about 2−3 kcal/mol [6,7,8,9,10]. Usually, docking scores are proportional to ΔG values. This low accuracy of the ΔG or docking score has contributed to a low success rate of computer-aided drug design. The limitations of the docking score are obvious. In statistical physics, the free energy is calculated from the partition function, which is based on a structural ensemble of numerous structures at a particular temperature. On the other hand, the conventional docking score is calculated from a single protein-compound complex structure.

Many reports have used molecular dynamics (MD) simulations to analyze protein-compound docking and to calculate the ΔG. Even if the protein-ligand complex structure is unknown, *ab-initio* MD docking simulations can be used to reveal the protein-ligand complex structures and the free energy landscapes [11,12,13,14]. In an explicit water model, if the protein-ligand complex structure is known, the binding free energy and the potential of mean force (PMF) along the dissociation path can be obtained using the filling potential (FP) method [15], the meta dynamics method [16,17], the MP-CAFEE method [18], the smooth-reaction path generation method [19] and Jarzynski’s method [20].

There have been several reports on the calculation of protein-ligand binding free energy by semi-empirical methods, since the *ab-initio* free energy calculation is still very time-consuming. The molecular-mechanical Poisson-Boltzman surface-area (MM-PBSA) method [21], the linear interaction energy (LIE) method [22] and the COMBINE method [23,24,25,26,27,28,29] have succeeded in reproducing the trends of ΔGs for single target proteins. These methods have been successful in practical use, but the parameters used in these methods must be optimized for each target protein.

We previously proposed a direct interaction approximation (DIA) method for the ΔG estimation [30]. This method estimates the ΔG value based on the direct interaction between the protein and the ligand just as in the COMBINE method, but the weighted parameters for residues are set to fixed values as in the LIE method. In the current study, we modified the DIA method in order to use target-independent parameters. Since previous authors have introduced a ligand-entropy term in their docking studies [5,6], we also examined the ligand-entropy term. In addition, because the mobility of solvent water molecules has been analyzed in previous reports [31,32], and we also examined the effect of the solvent water mobility herein, but used a different method of analysis for this purpose.

## 2. Results and Discussion

The brief explanation of the previously developed direct interaction approximation (DIA) [30] is introduced in Section 2.1. The ligand-entropy term is the first additional term to the original DIA and it is introduced in Section 2.2. The stability of hydration shell of each residue is the second additional term to the original DIA and it is introduced in Section 2.3. The ligand-entropy term and the stability of hydration shell were examined by using the protein-ligand complex structures in Section 2.4. These additional two terms improved the accuracy and the physical consistency of the DIA model. These results showed that the trajectory average of the protein-ligand interaction improved the estimation of the protein-ligand binding free energy. In Section 2.5, we showed the trajectory average of the docking score can improve the binding free energy estimation and the consensus score of the DIA result and the docking score improved the correlation between the experimental and the calculated binding free energies.

### 2.1. Original Direct Interaction Approximation (DIA) Method

In our previous study, we developed the DIA method to estimate ΔG [30]. The fluctuation of the accessible surface area (ASA) or the dihedral angles of the system was introduced as the entropy term of the ΔG value, and the estimation accuracy reached 1.5 kcal/mol for several tens protein-compound complex structures. Here, we will explain the DIA method briefly. In the original DIA method without a direct solvent effect (DIAV), the ΔG value is estimated as follows [30]:

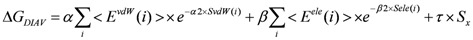
(1)
where E^vdW^(i) and E^ele^(i) are the vdW and electrostatic interactions between the i-th residue of the protein and the ligand, respectively. Svdw(i) and Sele(i) are the fluctuation of the E^vdW^(i) and E^ele^(i), respectively. The τ*S_x_ term represents the entropy of the system. S_x_ is the fluctuation of a property x. In the current study, S_x_ is the fluctuation of the accessible surface area (x = ASA) of the protein-ligand complex structure or the all dihedral angles (x = DIH) of the protein over the trajectory. There are five parameters: α, α2, β, β2, and τ.

To represent the van der Waals interaction and the hydrophobic interaction, a Lennard-Jones (LJ) 8-4-type function has been used instead of the LJ12-6 type function:

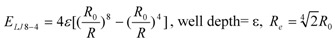
(2)
where R_e_ is the equilibrium distance. The R_e_ and the well depth values are set to the same values obtained from AMBER param99 [33] and the general AMBER force field (GAFF) [34]. The data sampling MD simulation is performed with the conventional AMBER force field (LJ 12-6 potential), and the DIA analysis is performed with Equation (2).

In the ligand-binding pocket, the effective dielectric constant (ε_eff_) should be different at each point, since the ε_eff_ values of proteins are 2−4 and the ε_eff_ of water is 78.5. The E^ele^(i) should be scaled by the ε_eff_. Therefore, the electrostatic interactions in the DIAV method were modified and we named the modified method as the direct interaction approximation with solvent (DIAS) method [30]:

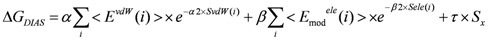
(3)
where E_mod_^ele^(i) is the E^ele^(i) value scaled by *ε_eff_*. The *ε_eff_* value could be calculated from the ratio between the electrostatic force calculated in the explicit water model and that in a vacuum as follows:

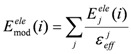
(4)
where E_j_^ele^(i) is the electrostatic interaction between the i-th residue and the j-th atom of the ligand in a vacuum. Here, the effective dielectric constant is given by:

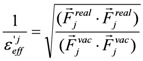
(5)
where F_j_^real^ and F_j_^vac^ are the electrostatic force acting on the j-th atom in the explicit water model and in a vacuum, respectively. The F^real^ and F^vac^ were calculated by the molecular dynamics simulation in the explicit water model and in a vacuum, respectively.

The scale factor 1/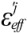 could be an unrealistically large value when the denominator of Equation (5) is nearly zero. Thus, we introduce a parameter x and the scale function as follows:

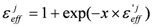
(6)


This parameter, 1/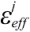, was used as the scale factor, and the previous study showed that the optimal value was 0.6 [30]. Note that the actual effective dielectric constant corresponds to 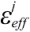/β.

### 2.2. Intra-Molecular Ligand-Entropy Term

In the current study, the entropy change of the ligand was taken into account in the ΔG estimation. The rotatable bonds of the ligand can freely rotate in its unbound form, and these bonds can be fixed into a single conformation in its bound form. Thus, the entropy of the ligand decreases during the protein−ligand binding process. We added the ligand-entropy term (TΔS_ligand_) as follows [5,6]:

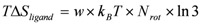
(7)
where N_rot_ is the number of rotatable bonds (single bonds between heavy atoms) in the non-ring part. The number of possible conformers is 3^Nrot^ without the ring part. Considering the intra-molecule atomic collision, the number of conformers can be less than 3^Nrot^, and so an additional parameter *w* is introduced.

First, the ligand entropy of the ring parts was examined. The number of conformers of a ring part was approximated by 2^(Nrot-ring -3)^ or 3^(Nrot-ring -3)^, where N_rot-ring_ is the number of rotatable bonds (single bonds between heavy atoms) in the ring parts, since the three-membered ring has only one conformer and the torsion angles of ring parts are restricted compared to the free rotation. We examined the importance of the ring-entropy term by multiple linear regression analysis of the data of 34 protein-ligand complexes. Consequently, the ring-entropy term did not improve the estimation accuracy of ΔG. Thus, in the current work, Equation (7) was used as the ligand-entropy term.

### 2.3. Hydration Effect of Each Residue of the Target Protein

In computer-aided drug design, crystal water molecules are often replaced by ligand atoms to design a high-activity compound [35]. This is an empirical procedure known among medicinal chemists. The amino-acid residues around the crystal water molecules should be important to the protein-ligand binding interaction (hot spot). To detect the hot spot, the mobility of water molecules is observed by MD simulations. In the current study, the MD simulation of apo protein in water was performed at room temperature, and the mobility of the water molecules was observed around the i-th residue.

The mobility of water is measured by the ligand exchange rate. In the current study, a residue-based ligand exchange rate for the i-th residue (<H_i_>) was introduced:

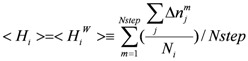
(8)


Here Δnm j is the number of water molecules exchanged at the m-th step of the sampled MD trajectory around the j-th atom. The j-th atom belongs to the i-th residue and N_i_ is the total number of atoms (including H atoms) of the i-th residue. Nstep is the total number of the sampled MD steps. The water molecule, whose distance to the j-th residue is less than 6 Å, is taken into account as the ligand of the j-th atom in Equation (8). Since the weight for each energy term of Equations (1) and (3) [exp(−α2xS_vdw_(i)) and exp(−β2xS_ele_(i))] corresponds to the probability, the weight for the i-th residue is a dimensionless parameter. We assume that the weight of the amino-acid residues is a function of <H_i_>, since it could be a measure of the stability of hydration shell around the i-th residue. The higher the value of <H_i_> is, the more important is the i-th residue is in the protein-ligand interaction. Thus, the weight of residues should be a monotonically increasing function of <H_i_>. We apply exp(γ<H_i_>) as a simple function for approximating the weight, where γ is a positive parameter. In the current study, the trajectory was sampled every 2 psec and the minimum, maximum and average values of <H_i_> were 0.0, 0.16, and 0.042, respectively. These values correspond to 1, 0.26, and 0.96 of the exp(γ<H_i_>) values. The average <H_i_> values in the ligand binding pocket and on the protein surface were 0.0478 and 0.0488, respectively. The ratios of the residue with <H_i_> less than the average <H_i_> value were 54% and 48% in the ligand binding pocket and on the protein surface, respectively.

Water molecules in the bottom of the pocket hardly move and the contact number of the bottom of the pocket should be large. Thus there is a correlation between the water mobility and the contact number. The contact number is the number of atoms (protein atoms only, excluding the solvent and ligand atoms), whose distances from the atom in question are less than 6 Å. In this assumption, the <H_i_> value for the i-th residue is estimated instead of Equation (8) as follows:

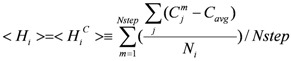
(9)


Here C_j_^m^ is the contact number of the j-th atom at the m-th step of the sampled MD trajectory and C_avg_ is the average value of C_j_^m^. The j-th atom belongs to the i-th residue and N_i_ is the total number of atoms of the i-th residue. In the current study, the correlation coefficient between the <
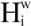
> and <
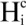
> was 0.32. The average (C_avg_), minimum and maximum <
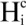
> values were 73.51, 0, and 106.2, respectively.

### 2.4. ΔG Estimation by the DIA Method

In the current study, we used the following 6 models to examine the ligand-entropy term and the hydration effect of residues.

Model 1: The original DIAV model described in Equation (1). Here, α2 and β2 were set to zero.

Model 2: The original DIAS model described in Equation (3).

Model 3: The DIAV_L model, where the ligand-entropy term is added to the original DIAV model:

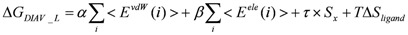
(10)


Model 4: The DIAV_W model, where the weight of each residue is calculated from the hydration solvent water. Here <H_i_> = <H_i_^w^> as in Equation (8):

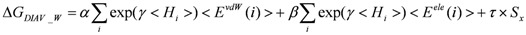
(11)


Model 5: The DIAV_LW model. Here, the ligand-entropy term and the weight for each residue are added to the original DIAV model. The weight for each residue is calculated from the hydration solvent water. Here <H_i_> = <H_i_^w^> as in Equation (8):

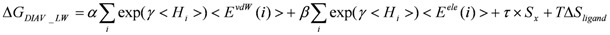
(12)


Model 6: The DIAV_LC model. The weight for each residue is estimated based on the contact number. The model equation is Equation (12) with the relation <H_i_> = < 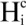 > as in Equation (9).

Table 1 shows the computational average error and the correlation coefficient between the experimental values and the values calculated by these six models. The results were obtained by leave-one-out cross-validation tests. In the leave-one-out cross-validation test, one data is selected as the test data that is to be predicted and the other data are used as the teaching data to generate the prediction model equation. The test data is selected one after another in the given data set until all data are selected as the test data. The property x of Sx (entropy term) was fixed to x = DIH (the fluctuation of the dihedral angles), since the ΔG accuracy obtained by x = DIH was better than that obtained by x = ASA (the fluctuation of the accessible surface area). The γ values were optimized to minimize the ΔG estimation error, and these values were set to −6.115 and 0.00613 for the DIAV_LW and DIAV_LC methods, respectively.

Comparing the average error obtained by the DIAV and DIAV_L models, the consideration of the ligand-entropy term improved the accuracy. In addition, comparing the average error obtained by the DIAV and DIAV_W models, the consideration of the weight of the residues improved the accuracy. The combination of both the ligand-entropy term and the weight of the residues improved the accuracy (DIAV_LW). Among the six models examined, the DIAV_LW model showed the best accuracy and the DIAV_C model showed the second best accuracy. The accuracy of the DIAV_L model was almost the same as that of the DIAS model. Since the number of parameters of the DIAV_L model was smaller than that of the DIAS model, the second best model should be the DIAV_L model.

**Figure 1:**
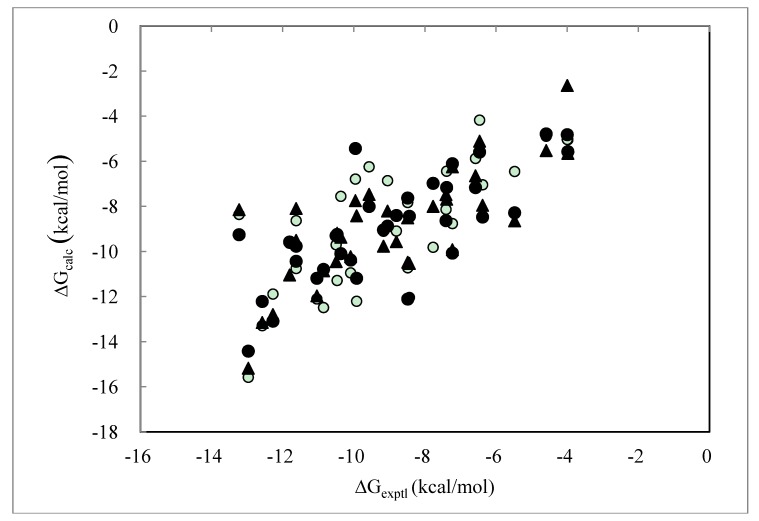
Cross-validation results obtained by the DIAV, DIAV_LW and DIAV_LC methods: The experimental data (ΔG_exptl_) and the calculated values (ΔG_calc_). Open circles, filled circles and filled triangles represent the results obtained by the DIAV, DIAV_LW and DIAV_LC methods, respectively.

Figure 1 shows the correlation between the experimental and calculated ΔG values obtained by the DIAV, DIAV_LW and DIAV_LC methods. These values were obtained by the molecular dynamics simulation starting from the experimentally determined protein-ligand complex structures. The computational detail was described in the data preparation section. It is clear that the DIAV_LW/DIAV_LC methods gave a better correlation than the DIAV method. 

**Table 1:** Estimated binding free energies by several models and the experimental value (*ΔG_exptl_*).

PDB ID	*ΔG_exptl_*	*ΔG_DIAV_*	*ΔG_DIAS_*	*ΔG_DIAV_L_*	*ΔG_DIAV_W_*	*ΔG_DIAV_LW_*	*ΔG_DIAV_LC_*
		Equation (1)	Equation (3)	Equation (10)	Equation (11)	Equation (12)	Equation (9,12)
1abf	−7.39	−6.44	−7.46	−7.33	−6.35	−7.16	−7.68
1apu	−10.50	−9.70	−10.70	−9.00	−9.70	−9.30	−10.45
1dbb	−12.27	−11.89	−11.25	−12.08	−11.67	−13.09	−12.80
1dbj	−10.47	−11.28	−10.39	−11.35	−11.07	−9.24	−9.19
1dog	−5.48	−6.45	−8.58	−8.00	−6.38	−8.28	−8.64
1dwb	−3.98	−5.04	−5.16	−5.24	−4.92	−5.57	−5.65
1epo	−10.85	−12.49	−12.35	−13.13	−12.53	−10.79	−10.85
1etr	−10.09	−10.95	−9.86	−9.90	−10.87	−10.38	−10.23
1ets	−11.62	−10.75	−10.21	−10.48	−10.62	−10.43	−9.51
1ett	−8.44	−12.04	−10.87	−10.42	−11.76	−8.44	−10.53
1hpv	−12.57	−13.29	−13.32	−12.78	−13.33	−12.22	−13.15
1hsl	−9.96	−6.79	−7.86	−7.26	−6.74	−5.43	−7.74
1htf	−11.04	−12.10	−10.45	−11.48	−12.13	−11.19	−11.97
1hvr	−12.97	−15.58	−14.97	−15.33	−15.63	−14.42	−15.18
1nsd	−7.23	−8.76	−9.21	−9.19	−8.65	−10.07	−9.92
1pgp	−7.77	−9.81	−9.10	−8.99	−9.56	−6.98	−8.00
1phg	−11.81	−9.63	−9.57	−10.59	−9.53	−9.58	−11.04
1ppc	−8.80	−9.09	−8.55	−9.44	−9.10	−8.40	−9.56
1pph	−8.49	−7.83	−7.46	−8.13	−7.81	−7.63	−8.51
1rbp	−9.17	−9.10	−9.62	−9.74	−9.11	−9.04	−9.76
1tng	−4.00	−5.03	−5.39	−5.48	−4.98	−4.82	−2.64
1tnh	−4.59	−4.89	−5.53	−5.26	−4.83	−4.78	−5.52
1ulb	−7.23	−6.18	−5.90	−6.06	−5.99	−6.10	−6.25
2cgr	−9.92	−12.21	−11.20	−11.16	−11.99	−11.19	−8.41
2gbp	−10.36	−7.55	−9.23	−8.63	−7.45	−10.09	−9.37
2ifb	−7.41	−8.13	−7.89	−7.08	−8.15	−8.63	−7.48
2phh	−6.38	−7.04	−7.57	−7.31	−6.83	−8.47	−7.95
2r04	−8.48	−10.72	−10.58	−10.29	−10.71	−12.11	−10.48
2tsc	−11.62	−8.63	−9.97	−8.90	−8.75	−9.76	−8.09
2ypi	−6.58	−5.87	−6.53	−6.20	−5.76	−7.16	−6.64
3ptb	−6.46	−4.17	−4.75	−4.75	−4.12	−5.59	−5.11
4dfr	−13.23	−8.35	−7.96	−8.16	−8.36	−9.25	−8.14
5abp	−9.05	−6.86	−8.12	−7.46	−6.77	−8.87	−8.21
Average Error		1.58	1.36	1.39	1.48	1.26	1.31
SD ^a^		1.88	1.66	1.68	1.86	1.70	1.72
Correlation coefficient		0.59	0.75	0.76	0.76	0.75	0.75
Average Error (MLR) ^b^		1.42	1.23	1.23	1.32	1.13	1.17

a: standard deviation of the difference between calculated and measured binding free energy. b: We also applied the multiple linear regression (MLR) to the 34 protein-ligand complex data. “Average Error (MLR)” is the averaged error obtained by the MLR. The error of the MLR is always smaller than the error obtained by the cross−validation test.

Table 2 shows the average, deviation, and minimum and maximum values of the optimized parameters (α, β, τ and w) of Models 1−6 of the 34 cross-validation tests. The % of the negative values is also summarized. The other parameters, *i.e.*, α2, β2, and γ, are omitted. The smaller the deviation of the parameter is, the less dependent on the target protein the model is. In particular, the sign of parameter β is important. Negative values of β are physically unreasonable. Namely, negative β implies that repulsive ligand-protein interactions stabilize the free energy of binding. In the DIAS and the DIAV models, the β value was negative in 2.86% of the cases (one model among total 34 cross-validation test models). In contrast, the average α, τ and w values were almost identical among the DIAV_L, DIAV_LW, DIAV_LC and DIAS models. The consideration of the ligand-entropy term (DIAV_L model) slightly improved the problem of the negative β parameter. In addition, the weight of residues (DIAV_W model) also slightly improved the problem of the negative β parameter. In the DIAV_LW and DIAV_LC models, all β parameters were positive in the 34 cross-validation tests.

Among these six models, the deviation of the DIAV_LW model was the smallest. Considering the average error and the deviation of the parameters, the DIAV_LW model was the best of the 6 models and the DIAV_LC model is the second best. In the drug design, the prediction accuracy of unknown compounds for a new target protein is more important than the regression of the activities of known active compounds for a known target. Thus, the parameters of the computational model must not depend on the target proteins. From this point of view, the DIAV_LW or DIAV_LC model is desirable.

**Table 2:** Summary of parameters determined by the cross-validation tests.

**DIAV**	**α**	**β**	**τ**	**w**
Average	0.0341719	0.0017533	−0.0002198	0.0000000
Deviation (σ)	0.0005495	0.0011874	0.0000087	0.0000000
Min	0.0323511	−0.0038807	−0.0002438	0.0000000
Max	0.0357564	0.0049798	−0.0002027	0.0000000
Negative value	0.0000000	0.0285714	1.0000000	0.0000000
**DIAV_L**	**α**	**β**	**τ**	**w**
Average	0.0370196	0.0029651	−0.0000050	0.1749169
Deviation (σ)	0.0007599	0.0008450	0.0000002	0.0190521
Min	0.0350933	−0.0000641	−0.0000054	0.1132383
Max	0.0396249	0.0047204	−0.0000045	0.2325974
Negative value	0.0000000	0.0285714	1.0000000	0.0000000
**DIAV_W**	**α**	**β**	**τ**	**w**
Average	0.0346823	0.0021929	−0.0002054	0.0000000
Deviation (σ)	0.0005388	0.0011036	0.0000083	0.0000000
Min	0.0329273	−0.0030242	−0.0002290	0.0000000
Max	0.0362899	0.0050095	−0.0001878	0.0000000
Negative value	0.0000000	0.0285714	1.0000000	0.0000000
**DIAV_LW**	**α**	**β**	**τ**	**w**
Average	0.0413163	0.0062033	−0. 0000067	0.1536118
Deviation (σ)	0.0007382	0.0007907	0. 0000002	0.0140040
Min	0.0392677	0.0034216	−0. 0000071	0.1254044
Max	0.0434480	0.0087162	−0. 0000063	0.1944447
Negative value	0.0000000	0.0000000	1.0000000	0.0000000
**DIAV_LC**	**α**	**β**	**τ**	**w**
Average	0.0343046	0.0042958	−0.0000070	0.1143295
Deviation (σ)	0.0006129	0.0011950	0.0000002	0.0097216
Min	0.0321378	0.0002835	−0.0000076	0.0942835
Max	0.0363001	0.0090566	−0.0000067	0.1414504
Negative value	0.0000000	0. 0000000	1.0000000	0.0000000
**DIAS**	**α**	**β**	**τ**	**w**
Average	0.0392333	0.0030804	−0.0000053	0.0000000
Deviation (σ)	0.0005573	0.0010426	0.0000002	0.0000000
Min	0.0375654	−0.0017236	−0.0000056	0.0000000
Max	0.0409116	0.0055269	−0.0000049	0.0000000
Negative value	0.0000000	0.0285714	1.0000000	0.0000000

The consideration of the ligand entropy and the weight of each residue did not improve the DIAS model. In the DIAS model, the weight of each residue is already considered by using the parameters α2 and β2. Thus, the newly introduced weight with the γ parameter would count the weight of the residue twice. Considering the number of parameters (α, β, τ, γ and w in the DIAV_LV/DIAV_LC models, and α, β, τ, α2, β2 and γ in the DIAS model), the DIAV_LW/DIAV_LC models have a smaller number of parameters than the DIAS model. Since a model with a small number of parameters should, in principle, be better than that with a large number of parameters, the DIAV_LW/DIAV_LC models are better than the DIAS model.

**Figure 2:**
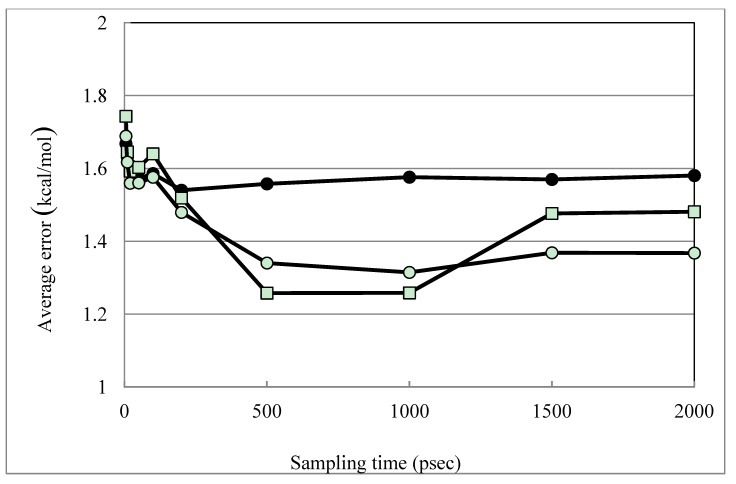
Time dependence of the DIAV, DIAV_LW and DIAV_LC results. Filled circles, open squares and open circles represent the results obtained by the DIAV, DIAV_LW and DIAV_LC methods, respectively. The averaged error is plotted vs. the sampling-time length.

The trajectory dependence of the models was examined. The above results were obtained from the 2-nsec trajectories. Figure 2 shows the time dependence of the DIAV, DIAV_LW and DIAV_LC results. When the 1-nsec trajectories were used, the results were slightly worse than those shown above, but the difference was not statistically significant. In the DIAV model, the β value was negative in 5.71% of the cases (1ett and 1hsl). In the DIAS, DIAV_L and the DIAV_W models, the β value was negative in 2.86% of the cases (1ett in the all models) and no negative β value was observed in the DIAV_LW/DIAV_LC models, just as in the above results. The 1ett structure is thrombin, but the other thrombin structures (1etr, 1ets) did not show the problem. Currently, the reason of the problem is unclear.

We also estimated the binding free energies of non-active compounds, since evaluation of the binding free energies of the both active and non-active compounds are essential in practical use. We docked three GPCR (G-protein coupled receptor: membrane protein) ligands to the proteins and calculated the binding free energies of them by the DIAV_L method. These compounds were alprenolol (a β-adrenergic inverse agonist), fenoterol (a β-adrenergic inverse agonist) and cetirizine (H_1_ receptor inverse agonist). They are non-active compounds of the proteins, because there are no GPCR in the target proteins. The condition of the MD simulation was the same as those used in Table 1. The parameters of the DIAV_L method were determined for the MLR of the all 34 proteins used in Table 1. The results are summarized in Table 3. In some cases, the pocket sizes of the ligand-binding sites of the proteins were too small for the GPCR ligands to be docked, and so MD simulations were not possible for them. Obviously, such ligands should be non-active compounds. The average values of the estimated binding free energies for the non-active compounds were weaker than those of the original ligands. In all 19 cases, the ΔGs of alprenolol were weaker than those of the original ligand. The ΔGs of fenoterol and cetirizine were weaker than those of the original ligand in 69% and 56% cases, respectively. For some proteins, feneterol and cetirizine show stronger affinities estimated than those for the original ligands, but most of their absolute bindings are weak.

**Table 3:** The binding free energies estimated for the non-active compounds (kcal/mol).

PDB ID	*ΔG_exptl_*	*ΔG_DIAV_L_*						
		Original ligand	Alprenolol	Difference ^a^	Fenoterol	Difference ^a^	Cetirizine	Difference ^a^
1abe2	−9.57	−8.06	−6.85	−1.22	−8.21	0.14	−9.28	1.22
1abf1	−7.39	−8.40	−6.13	−2.27	−6.72	−1.68	−7.93	−0.47
1apu	−10.50	−11.63	−2.77	−8.86	−4.50	−7.12	−5.69	−5.93
1cbx	−8.65	−8.89	−5.84	−3.04	−7.51	−1.38	−8.30	−0.58
1dog	−5.48	−9.05	−5.18	−3.87	−7.75	−1.30	−5.08	−3.97
1dwb	−3.98	−5.45	−5.44	−0.01	−6.56	1.11	−8.24	2.80
1ebg	−14.76	−6.74	0.00	−6.74	0.00	−6.74	0.00	−6.74
1epo	−10.85	−14.42	−5.64	−8.78	−7.30	−7.12	−8.49	−5.93
1rbp	−9.17	−8.76	N.D.^b^	N.D. ^b^	N.D. ^b^	N.D. ^b^	−8.69	−0.08
1stp	−18.27	−6.59	N.D. ^b^	N.D. ^b^	N.D. ^b^	N.D. ^b^	−5.96	−0.63
1tnh	−4.59	−5.59	−4.39	−1.20	−5.62	0.03	−6.13	0.54
1ulb	−7.23	−6.19	−5.45	−0.74	−6.23	0.04	−8.98	2.79
2gbp	−10.36	−10.14	−7.16	−2.98	−8.74	−1.40	−10.24	0.11
2ifb	−7.41	−8.60	−5.81	−2.79	−7.09	−1.51	−9.01	0.41
2tsc	−11.62	−8.23	−5.68	−2.55	−6.48	−1.75	−8.69	0.47
2ypi	−6.58	−6.92	−4.68	−2.24	N.D. ^b^	N.D. ^b^	N.D. ^b^	N.D. ^b^
3ptb	−6.46	−4.96	−4.49	−0.48	−5.89	0.93	−5.64	0.68
4dfr	−13.22	−8.42	−5.16	−3.26	−5.64	−2.79	−6.66	−1.76
6cpa	−15.71	−11.68	−6.82	−4.86	−7.77	−3.91	−9.75	−1.93
Average	−9.57	−8.35	−5.15	−3.29	−6.38	−2.15	−7.38	−1.06

a: the energy difference between the calculated ΔG of the original ligand and the non-active ligand.b: Not Determined, because the pocket sizes of the ligand−binding sites of the proteins were too small for these ligands to be docked, and so MD simulations were not possible for them.

The whole protein set included four thrombins, three HIV-1 proteases and five trypsins. We have examined the Spearman’s rank correlations for these ligands of the same target proteins. The parameters of the DIAV_L, DIAV_LW and DIAV_LC methods were determined based on the 22 proteins excluding these 12 target proteins. The ΔGs, error of the ΔGs, and the correlation coefficients are summarized in Table 4. The total number of HIV-1 proteases was 11, since we added eight new data points. These results obtained by the DIAV_L, DIAV_LW and DIAV_LC methods are similar to each other. The trends of the ΔGs are almost correctly reproduced.

**Table 4:** ΔG values (kcal/mol) of the same target proteins and Spearman’s rank correlations.

Thrombin	*ΔG_exptl_*	*ΔG_DIAV_L_*	Error	*ΔG_DIAV_LW_*	Error	*ΔG_DIAV_LC_*	Error
1dwb	−3.98	−5.15	1.17	−5.02	1.04	−5.57	1.59
1ett	−8.44	−9.9	1.46	−9.74	1.31	−9.81	1.37
1etr	−10.09	−9.9	0.19	−9.89	0.2	−10.22	0.13
1ets	−11.62	−10.9	0.72	−10.76	0.86	−10.46	1.16
Averaged error (kcal/mol)	-	-	0.89	-	0.85	-	1.06
SD^a^	*-*	*-*	1.01	*-*	0.95	*-*	1.20
Correlation coefficient	*-*	*-*	0.97	*-*	0.97	*-*	0.96
Spearman’s rank correlation	*-*	*-*	1	*-*	1	*-*	1
**HIV-1 Protease**	***ΔG_exptl_***	***ΔG_DIAV_L_***	**Error**	***ΔG_DIAV_LW_***	**Error**	***ΔG_DIAV_LC_***	**Error**
1k6p	−8.84	−11.71	2.87	−11.74	2.90	−11.78	2.94
1ajv	−10.59	−10.36	0.23	−10.39	0.20	−10.13	0.46
1ajx	−10.86	−9.89	0.97	−9.91	0.95	−9.68	1.18
1hih	−10.97	−11.67	0.70	−11.67	0.70	−11.73	0.76
1htf	−11.04	−11.57	0.53	−11.59	0.55	−11.86	0.82
1aaq	−11.45	−13.15	1.70	−13.13	1.68	−12.96	1.51
1hpv	−12.57	−12.79	0.22	−12.87	0.30	−13.06	0.49
1hvr	−12.97	−14.79	1.82	−14.93	1.96	−14.65	1.68
1hvk	−13.79	−13.63	0.16	−13.65	0.14	−13.70	0.09
1vj	−14.62	−12.82	1.80	−12.85	1.77	−12.89	1.73
1dif	−14.63	−13.76	0.87	−13.77	0.86	−13.82	0.81
Averaged error (kcal/mol)	-	-	1.08	-	1.09	-	1.13
SD^a^	-	-	1.36	-	1.37	-	1.37
Correlation coefficient	-	-	0.68	-	0.67	-	0.68
Spearman’s rank correlation	-	-	0.78	-	0.75	-	0.81
**Trypsin**	***ΔG_exptl_***	***ΔG_DIAV_L_***	**Error**	***ΔG_DIAV_LW_***	**Error**	***ΔG_DIAV_LC_***	**Error**
1tng	−4.00	−5.45	1.45	−5.36	1.37	−2.69	1.31
1tnh	−4.59	−5.29	0.70	−5.20	0.61	−5.50	0.91
3ptb	−6.46	−4.92	1.54	−4.83	1.63	−5.15	1.31
1pph	−8.48	−8.32	0.16	−8.30	0.18	−8.51	0.02
1ppc	−8.80	−9.32	0.52	−9.31	0.51	−9.53	0.72
Averaged error (kcal/mol)	-	-	0.88	-	0.86	-	0.86
SD^a^	-	-	1.03	-	1.02	-	0.98
Correlation coefficient	-	-	0.86	-	0.86	-	0.93
Spearman’s rank correlation	-	-	0.60	-	0.60	-	0.90

a: standard deviation of the difference between calculated and measured binding free energy.

### 2.5. Consensus Score with the Trajectory Average of the Docking Score

Next, we examined the trajectory average of the docking score. The Sievgene protein-compound docking program was used to calculate the protein-ligand docking score [7]. The trajectory average improved the correlation between the experimental binding free energy and the averaged docking score. Namely, the correlation coefficients between the experimental binding free energy and the averaged docking score were 0.751 and 0.745, with and without the trajectory average, respectively. The actual docking scores calculated by three different programs were summarized in the supplementary data. On the contrary, the DIAV_L (R = 0.76) and DIAV_LW (R = 0.78) methods gave R = 0.76 and 0.78, respectively, slightly better than those for the averaged docking scores. However, the differences between their estimated binding free energies and the experimental ones were 1.39 kcal/mol and 1.33 kcal/mol, respectively. They are much smaller than those by the averaged docking scores, 1.63 kcal/mol and 1.89 kcal/mol with and without trajectory average, respectively. We must note that the results should strongly depend on the ensemble generated by the MD simulation [36]. Our method should be applied the correct protein-ligand complex structures or the protein-ligand complex structures must be the equilibrium states, otherwise the calculated ΔG values should drift depending on the simulation time.

The consensus score of the DIA model and the docking score was also examined. The simple sum of the ΔG value obtained by the DIAV_LW and Sievgene docking score gave a correlation coefficient between the consensus score and the experimental ΔG of 0.796. The simple sum of the ΔG value obtained by the DIAV_LC and Sievgene docking score gave a correlation coefficient between the consensus score and the experimental ΔG of 0.782. Thus, the consensus score worked well and it slightly improved the ΔG estimation.

Recently, a machine-learning approach was applied to improve the docking score. Such new method showed the ΔG standard deviation (SD) error of 1.5 kcal/mol and the correlation coefficients between the experimental binding free energy and the docking score reached 0.76 based on a single structure [37]. This result is better than our current result (R = 0.75−0.76, SD = 1.7–1.8 kcal/mol; see Table 1), however the used protein-ligand datasets were different to each other. The accuracy of the other docking score could be improved considering the suitable ensemble of the protein-ligand complex structures.

## 3. Method: The Docking Score Calculation

A protein-compound docking simulation was performed by the program Sievgene, which is a protein-ligand flexible docking program for *in silico* drug screening [7]. This program generates many conformers (100 conformers by default) for each compound and keeps the target protein structure rigid, but with soft interaction forces adapting its slight structural change to some extent. The Sievgene scoring function was designed to consider the structural change of the target protein. In the inner region of the target protein, the protein is approximated as an elastic body, while the atomic pair-wise scoring function is applied in the outer region of the target protein. This docking program was developed with a performance yielding about 50% of the reconstructed complexes at a distance of less than 2 Å RMSD for the 132 complexed receptors with the compounds in PDB. The results predicted by our program were almost the same as those predicted by other docking programs [7]. The docking score (*H_dock_*) to estimate the protein-ligand binding free energy was determined as:

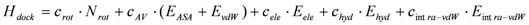
(15)
where *N_rot_*, *E_ASA_*, *E_vdW_*, *E_ele_*, *E_hyd_*, and *E_intra-vdW_* represent the number of rotatable bonds of the ligand molecule, the hydrophobic energy due to the accessible surface area, the vdW energy, the protein-ligand Coulombic potential, the hydrogen bond energy, and the intra-molecular vdW energy of the ligand for Sievgene [7]. Also, *c_rot_*, *c_AV_*, *c_ele_*, *c_hyd_*, and *c_intra-vdW_* are the optimized coefficients for each energy term. For each atom type, the sum of *E_ASA_* and *E_vdW_* gives a grid potential, and both energy terms are always simultaneously calculated. Thus, these two terms share the same coefficient, *c_AV_*. Sievgene utilizes the grid potential to calculate each energy term except for the intra-molecular interactions. In this study, a mesh size of 60 × 60 × 60 was adopted.

## 4. Data Preparation

To determine the coefficients for the *ΔG* scores for several current models, we performed a protein-ligand docking simulation based on the known complex structures registered in the Protein Data Bank. The data and the procedure were almost the same as those used in the previous study [30]. Here, 34 complexes accompanied by the experimental binding free-energy values were selected from the database that was used to determine the ΔG scores of the PRO_LEADS [6]. The PDB identifiers, the names (protein names and ligand names), the molecular weights (MW), the number of hydrogen bond acceptors (HA) and the number of hydrogen bond donors (HD) of ligands are summarized in Table 5. There was no peptide-like compound. The MWs were distributed from 114 to 606 Da. To assess the ligand diversity, we calculated the average Tanimoto index and the standard deviation of the all 34 ligands × all 34 ligands by using Maximum Common Substructure (MCS) method [38]. The average Tanimoto index and the standard deviation were 0.29 and 0.19, respectively. These values showed that the used ligand molecules were diverse. In the test dataset, the metalloproteins were removed from the present analysis. Metal atoms (Zn and Fe atoms) formed covalent bonds with O and S atoms of the ligands, and the classical force field that we applied could not represent the covalent bond. Thus, the present method cannot calculate ΔG values for metalloproteins with high precision.

The structural ensembles generated from the PDB structure given by MD in explicit water were prepared as follows. All target proteins were prepared with ligands (forming a protein-ligand complex structure). In the previous study, all metal atoms in the systems were removed, since the target proteins were not metalloproteases. Some non-metalloproteins include metal atoms those bind to the proteins. In the current study, all metal atoms of the PDB files were included in the MD simulations. The force fields and the charges of the protein atoms originated from AMBER parm99 [36]. The atomic charge of each ligand was determined by the restricted electrostatic point charge (RESP) procedure using HF/6-31G*-level quantum chemical calculations [39]. We used Gaussian98 to perform the quantum chemical calculations [40]. The initial coordinates of protein and ligand molecule of each data were fixed to the experimentally determined coordinates. The whole structure of each protein was embedded in a sphere of TIP3P [41] water molecules (CAP water), including ion particles of 0.1% Na^+^ and Cl^−^, in order to neutralize the total charge of the systems. The center of the sphere was set at the mass center of the protein. The shortest distance between the protein atom and the CAP sphere wall was set to 10 Å.

**Table 5:** List of the proteins and ligands used.

PDB ID	Protein	Ligand	MW	HA	HD
1abe	l-arabinose-binding protein	l-arabinose	150.1	5	4
1abf	l-arabinose-binding protein	d-fucose	161.2	5	4
1apu	acid proteinase (penicillopepsin)	pepstatin	485.7	6	4
1dbb	Fab' fragment	progesterone	314.5	2	0
1dbj	Fab' fragment	aetiocholanolone	290.4	2	1
1dog	glucoamylase	deoxynojirimycin	163.2	4	5
1dwb	thrombin	benzamidine	120.2	0	2
1epo	endothia aspartic proteinase	n-carbonylmorpholine	131.1	5	6
1etr	thrombin	MQPA	509.2	5	5
1ets	thrombin	NAPAP	522.6	4	4
1ett	thrombin	4-tapap	429.6	3	3
1hpv	HIV-1 protease	amprenavir	505.6	6	3
1hsl	Histidine-binding protein	Histidine	156.2	3	2
1htf	HIV-1 protease	GR126045	574.7	4	5
1hvr	HIV-1 protease	XK263	606.8	3	2
1nsd	neuraminidase	neuraminic acid	290.2	8	5
1pgp	6-phosphogluconate dehydrogenase	6-phosphogluconic acid	276.1	10	4
1phg	cytochrome P450	metyrapone	226.3	3	0
1ppc	trypsin	Napap	533.6	4	4
1pph	trypsin	3-Tapap	429.6	3	3
1rbp	retinol-binding protein	retinol	286.5	1	1
1tng	trypsin	aminomethylcyclohexane	114.2	0	1
1tnh	trypsin	4-fluorobenzylamine	126.2	0	1
1ulb	purine nucleoside phosphorylase	guanine	151.1	3	3
2cgr	Igg2b (KAPPA) Fab fragment	guanidineacetic acid	384.4	3	3
2gbp	d-galactose / D-glucose-binding protein	d-glucose	180.2	6	5
2ifb	intestinal fatty acid binding protein	palmitic acid	256.4	2	0
2phh	p-hydroxybenzoate hydroxylase	p-hydroxybenzoate	138.1	3	1
2r04	rhinovirus 14 (HRV14)	W71	342.4	5	0
2tsc	thymidylate synthase	10-propargyl-5,8-dideazafolic acid	477.5	7	3
2ypi	triose phosphate isomerase	2-phosphoglycolate	156.0	6	0
3ptb	trypsin	benzamidine	120.2	0	2
4dfr	dihydrofolate reductase	methotrexate	454.4	9	3
5abp	l-arabinose-binding protein	d-galactose	180.2	6	5

MW: Molecular weight (Da); HA: Number of hydrogen bond acceptors; HD: Number of hydrogen bond donors.

Before an MD calculation was performed for the entire system, an MD calculation for only the solvent parts (solvent water and counter ions) was performed with the protein, ligand, and metal ion coordinates fixed, so as to bring the solvent parts sufficiently close to an equilibrium state. The SHAKE method was used to constrain covalent bonds between heavy and hydrogen atoms in any molecule in the system [42]. MD simulations of the entire system were performed using 2.0 fs time steps with the temperature set at 310 K; the fast multipole method [43] was used to calculate the Coulombic interaction. The cutoff distance of the van der Waals interaction was 12.0 Å. The MD simulations were performed by using cosgene/myPresto [15]. After equilibration steps of 1,000 ps, the protein coordinates were sampled every 2 ps. Finally, we obtained 1,000 structures for each target protein in the 2,000 ps production run. The software program myPresto version 4 (http://presto.protein.osaka-u.ac.jp/myPresto4/index_e.html) was used for the simulation. The 2-nsec MD simulations cost average 79 h (max 229 h) using 4-core parallel computation on intel Xeon 5600. The trajectory analysis for the DIA method cost average 580 second using single core on intel Xeon 5600.

## 5. Conclusions

We have developed new computational models for protein-ligand binding free energy estimation. The DIAV_LC and DIAV_LW models were based on the trajectory average of the protein-ligand interaction with the ligand-entropy term. The mobility of the water molecules in the ligand-binding pocket was used to calculate the weight of the each residue-ligand interaction of the target protein. The interactions of residues around the low-mobility water were weighted comparing to the interactions of other residues. The consideration of the ligand entropy and the weight of the residues reduced the target-protein dependence of the parameters of the DIA models and consequently the accuracy was improved. The average error of ΔG estimation was 1.3 kcal/mol and the correlation coefficient between the experimental values and the calculated values was 0.75, when the correct protein-ligand complex structures were provided. The trajectory average of the docking score improved the correlation between the docking score and the experimental ΔG values. In addition, the simple sum of the ΔG value obtained by the DIAV_LW/DIAV_LC and a docking score showed the correlation coefficient between the consensus score and the experimental ΔG of 0.8. Thus, the consensus score worked well, and it slightly improved the ΔG estimation.

## Supplementary Files

Supplementary File 1 Supplementary data (PDF, 60 KB)
