# Supplementary material for: Improved Estimation of Protein-Ligand Binding Free Energy by Using the Ligand-Entropy and Mobility of Water Molecules
Source: Pharmaceuticals (Basel). 2013 Apr 26;6(5):604–22. doi: 10.3390/ph6050604 (PMC3817721; doi:10.3390/ph6050604)
Supplement: Supplementary File 1 — Supplementary data (PDF, 60 KB) [file pharmaceuticals-06-00604-s001.pdf]

Supplementary data: Estimated binding free energies by several models and the experimental value (ΔG_{exptl})

PDB ID	ΔG_{exptl}	ΔG_{DIAV} (eq. 1)	ΔG_{DIAS} (eq. 3)	ΔG_{DIAV_L} (eq. 9)	ΔG_{DIAV_W} (eq. 10)	ΔG_{DIAV_LW} (eq. 11)	ΔG_{DIAV_LC} (eq. 11)	PRO_LEADS[a]	*PSI-DOCK*[b]	*Sievgene*[c]
1abf	-7.39	-6.44	-7.46	-7.33	-6.35	-7.16	-7.68	-7.83	-8.34	-9.19
1apu	-10.50	-9.70	-10.70	-9.00	-9.70	-9.30	-10.45	-8.83	-9.88	-10.21
1dbb	-12.27	-11.89	-11.25	-12.08	-11.67	-13.09	-12.80	-9.79	-10.50	-7.91
1dbj	-10.47	-11.28	-10.39	-11.35	-11.07	-9.24	-9.19	-10.01	-10.83	-9.41
1dog	-5.48	-6.45	-8.58	-8.00	-6.38	-8.28	-8.64	-7.22	-8.68	-7.45
1dwb	-3.98	-5.04	-5.16	-5.24	-4.92	-5.57	-5.65	-5.33	-5.97	-5.09
1epo	-10.85	-12.49	-12.35	-13.13	-12.53	-10.79	-10.85	-11.32	-9.40	-13.26
1etr	-10.09	-10.95	-9.86	-9.90	-10.87	-10.38	-10.23	-9.92	-10.91	-11.01
1ets	-11.62	-10.75	-10.21	-10.48	-10.62	-10.43	-9.51	-11.25	-11.07	-13.11
1ett	-8.44	-12.04	-10.87	-10.42	-11.76	-8.44	-10.53	-8.31	-9.96	-10.87
1hpv	-12.57	-13.29	-13.32	-12.78	-13.33	-12.22	-13.15	-9.75	-9.53	-13.38
1hsl	-9.96	-6.79	-7.86	-7.26	-6.74	-5.43	-7.74	-7.47	-7.61	-8.30
1htf	-11.04	-12.10	-10.45	-11.48	-12.13	-11.19	-11.97	-11.74	-10.92	-12.15
1hvr	-12.97	-15.58	-14.97	-15.33	-15.63	-14.42	-15.18	-15.22	-12.49	-17.47
1nsd	-7.23	-8.76	-9.21	-9.19	-8.65	-10.07	-9.92	-7.02	-7.88	-11.68
1pgp	-7.77	-9.81	-9.10	-8.99	-9.56	-6.98	-8.00	-5.55	-8.48	-7.54
1phg	-11.81	-9.63	-9.57	-10.59	-9.53	-9.58	-11.04	-9.11	-7.51	-8.12
1ppc	-8.80	-9.09	-8.55	-9.44	-9.10	-8.40	-9.56	-9.43	-7.87	-11.00
1pph	-8.49	-7.83	-7.46	-8.13	-7.81	-7.63	-8.51	-8.90	-6.25	-8.98
1rbp	-9.17	-9.10	-9.62	-9.74	-9.11	-9.04	-9.76	-10.37	-11.83	-10.35
1tng	-4.00	-5.03	-5.39	-5.48	-4.98	-4.82	-2.64	-5.32	-5.12	-5.05

1tnh	-4.59	-4.89	-5.53	-5.26	-4.83	-4.78	-5.52	-5.14	-5.11	-5.15
1ulb	-7.23	-6.18	-5.90	-6.06	-5.99	-6.10	-6.25	-5.25	-7.28	-5.46
2cgr	-9.92	-12.21	-11.20	-11.16	-11.99	-11.19	-8.41	-8.62	-9.36	-10.57
2gbp	-10.36	-7.55	-9.23	-8.63	-7.45	-10.09	-9.37	-8.91	-9.05	-9.46
2ifb	-7.41	-8.13	-7.89	-7.08	-8.15	-8.63	-7.48	-10.41	-9.89	-8.41
2phh	-6.38	-7.04	-7.57	-7.31	-6.83	-8.47	-7.95	-7.68	-7.47	-6.35
2r04	-8.48	-10.72	-10.58	-10.29	-10.71	-12.11	-10.48	-10.34	-11.71	-10.55
2tsc	-11.62	-8.63	-9.97	-8.90	-8.75	-9.76	-8.09	-8.25	-10.10	-9.38
2ypi	-6.58	-5.87	-6.53	-6.20	-5.76	-7.16	-6.64	-5.41	-7.23	-5.59
3ptb	-6.46	-4.17	-4.75	-4.75	-4.12	-5.59	-5.11	-6.17	-5.82	-5.56
4dfr	-13.23	-8.35	-7.96	-8.16	-8.36	-9.25	-8.14	-9.39	-9.65	-12.03
5abp	-9.05	-6.86	-8.12	-7.46	-6.77	-8.87	-8.21	-7.80	-8.68	-9.67
Average Error		1.58	1.36	1.39	1.48	1.26	1.31	1.44	1.45	1.58
SD		1.88	1.66	1.68	1.86	1.70	1.72	1.76	1.82	1.97
Correlation coefficient		0.59	0.75	0.76	0.76	0.75	0.75	0.74	0.70	0.75
Average Error (MLR)[a]		1.42	1.23	1.23	1.32	1.13	1.17	1.44	1.45	1.58

a: Reference 6.

b: Pei, J.; Liu, Z.; Li, Q.; Yang, K.; Lai, L. PSI-DOCK: Towards highly efficient and accurate flexible ligand docking. *PROTEINS*. **2006**, 62, 934-946.

c: Reference 7.

The estimated values by PRO_LEADS, PSI-DOCK and sievgene are fitted values, while the values by the DIA methods are the cross validation test results.
